# Carbon Dot/Naphthalimide Based Ratiometric Fluorescence Biosensor for Hyaluronidase Detection

**DOI:** 10.3390/ma14051313

**Published:** 2021-03-09

**Authors:** Pushap Raj, Seon-yeong Lee, Tae Yoon Lee

**Affiliations:** 1 Department of Convergence System Engineering and Department of Biomedical Engineering, Chungnam National University, 99 Daehak-ro, Yuseong-gu, Daejeon 34134, Korea; pushap143@cnu.ac.kr; 2 Department of Technology Education, Chungnam National University, 99 Daehak-ro, Yuseong-gu, Daejeon 34134, Korea; t.sylee29@gmail.com

**Keywords:** fluorescence, hyaluronic acid, biosensor, hyaluronidase, naphthalimide

## Abstract

Bladder cancer is the leading cause of death in patients with genitourinary cancer. An elevated level of hyaluronidase (HAase) was found in bladder cancer, which acts as an important biomarker for the early diagnosis of bladder cancer. Hence, there is a need to develop a simple enzymatic assay for the early recognition of HAase. Herein, we report a simple, sensitive, and ratiometric fluorescence assay for HAase detection under physiological conditions. The fluorescence assay was constructed by the adsorption of cationic carbon dots and positively charged naphthalimide on negatively charged hyaluronic acid and the development of a Förster resonance energy transfer (FRET) mechanism from carbon dots to a naphthalimide fluorophores. The hyaluronidase enzyme cleaves the hyaluronic acid in this assay, and breaking down the FRET mechanism induces ratiometric changes. A detection limit of 0.09 U/mL was achieved, which is less than the HAase level found in normal human body fluids. Moreover, this assay may be used for diagnosing HAase-related diseases.

## 1. Introduction

Hyaluronic acid (HA) is a linear polysaccharide composed of D-glucuronic acid and N-acetyl-D-glucosamine linked through β (1–3) and β (1–4) glycosidic bonds [1,2]. It is an anionic, non-sulfated glycosaminoglycan produced by the plasma membrane and energetically stable biomolecules [3]. HA is a very important component of the extracellular matrix, and it directly regulates many biological activities, such as cell adhesion, migration, and proliferation [4,5,6]. Hyaluronidase (HAase) is an enzyme that can cleave the HA into small fragments [7]. Elevated levels of HAase are found in many types of cancer, including bladder cancer, prostate cancer, malignant melanoma, and brain cancer [8,9,10]. Consequently, it has an important role in clinical diagnosis and early therapy [11]. Bladder cancer is one of the most frequent malignancies and the second highest cause of death in genitourinary cancer patients annually [12,13]. Owing to the high levels of HAase in bladder cancer, HAase has emerged as a new biomarker for the early diagnosis of bladder cancer through the investigation of urinary HAase levels [14,15]. Therefore, it is important to develop a simple and ultrasensitive analytical method for the early detection of HAase. Various methods have been reported for the detection of HAase, such as turbidimetry, viscosimetry, colorimetry, and zymography [16,17,18,19,20]. However, these classical analytical methods have many shortcomings, such as specificity, sensitivity, time-consuming sample preparation, and inaccurate detection results. Immunoassays are another sensitive and specific analytical approach for the detection of HAase [21]. However, they require expensive antibodies and very tedious conjugation and washing steps, which restrict their wide applicability. Recently, fluorescence methods have gained considerable attention for biomolecule detection, owing to their high stability, sensitivity, and feasibility for quantification [22,23,24,25]. Based on the donor–acceptor design, numerous fluorescence molecular probes, quantum dots, and gold nanoparticles (AuNPs) have been synthesized for hyaluronidase monitoring [26,27]. HAase detection was achieved by the cleavage of the HA assembly, which leads to emission recovery; this was labeled with quantum dots, fluorescent dyes, gold nanoparticles, and up-conversion fluorescent nanoparticles (UCNP) [28,29,30]. These fluorescence probes have many limitations, such as the blue emission of carbon dots, which can be easily affected by background noise, the aggregation of AuNPs in high-salt biological media, tedious synthetic procedures, multiple non-specific adsorption interactions of molecular probes, and the covalent modification of HA, which are time-consuming and affect the specificity of the sensing probes. Additionally, most of the fluorescence sensors reported for HAase detection are single-wavelength emission, which is easily fluctuated by the external environment, such as temperature, external light, and concentration, thereby inducing false positive results. Yang et al. developed electrostatically-controlled ratiometric fluorescence probes for HAase monitoring [4]. The electrostatic interaction between positively charged fluorescent components (carbon dot as a donor and organic dye as an acceptor) and negatively charged HA develops a Förster resonance energy transfer (FRET) pair assay that exhibits a ratiometric response. Upon the enzymatic hydrolysis of HA, the FRET pair breakdown and ratiometric emission intensity diminish. Based on the same mechanism, fluorescein isothiocyanate (donor) and AuNPs (acceptor) were encapsulated in HA hydrogel [31]. A fluorescence signal was generated in the presence of HAase. Furthermore, Ma et al. developed a perylene (PDI)-based self-assembly for HAase detection [3]. The electrostatic interactions between negatively charged HA and positively charged perylene lead to aggregation-caused quenching. The enzymatic hydrolysis of HA leads to a disruption of the aggregation phenomenon and the recovery of the fluorescence intensity of pure PDI. These fluorescent probes exhibit appropriate simplicity and sensitivity for HAase recognition. However, molecular probes with higher stability and sensitivity are still required for the early diagnosis of cancer. Thus, the development of new fluorescence probes with high sensitivity, selectivity, and stability is of interest for biosensing applications [32]. Herein, we report a simple enzymatic fluorescence assay of negatively charged HA, super-cationic carbon dots, and positively charged naphthalimide fluorophores for HAase detection. Naphthalimide fluorophores are used, owing to their excellent quantum yield, easy synthesis, biocompatibility, water solubility, and tunable electronic properties [33,34,35]. Similarly, carbon dots are selected owing to their easy and low-cost synthesis, tunable fluorescence, and biocompatibility [36,37,38]. The overlap of the absorption and emission spectra of naphthalimide and carbon dots leads to the development of a FRET mechanism between both fluorophores. This FRET mechanism was controlled by HA and the HAase, which used for hyaluronidase monitoring. The advantage of this assay is that it is simple, highly sensitive, economically viable, and less time-consuming. Moreover, it shows a ratiometric response that is not altered by external factors, such as temperature, concentration, and external light.

## 2. Material and Methods

### 2.1. Chemicals and Reagents

All chemicals used in this study were of analytical grade. They were purchased from Sigma Aldrich Inc. (Merck KGaA, Darmstadt, Germany) and Alfa Aeser (Thermo Fisher Scientific Inc., Waltham, MA, USA) and used without further purification. The 4-aminonaphthalic-anhydride, phosphate buffered saline (PBS) buffer, hyaluronidase, bovine serum albumin (BSA), alkaline phosphatase (ALP), human serum albumin (HSA), glutathione (GSH), glucose, and cysteamine (Cys) were purchased from Sigma Aldrich Inc., and the HA was purchased from Acros Organics (Thermo Fisher Scientific Inc., Waltham, MA, USA). The methyliodide, 3-(dimethylamino)-1-propylamine, ethanol, methanol, spermidine trihydrochloride, CaCl_2_, NaCl, KCl, MgCl_2_, Zn(NO_3_)_2_, and Cu(NO_3_)_2_ were purchased from Alfa Aeser.

### 2.2. Instrumentation

^1^H nuclear magnetic resonance (NMR) and ^13^C NMR spectra were recorded on a 600 MHz NMR spectrometer (AVANCE III HD; Bruker Corp., Billerica, MA, USA). The chemical shift values were measured in ppm with respect to tetramethylsilane as an internal standard. The NMR spectra of the compounds were analyzed using TopSpin 4.0.8 software. The ultraviolet (UV)-visible absorption spectra were measured on a UV-visible absorption spectrophotometer (S-3100; Scinco Inc., Seoul, Korea). The samples were recorded in the absorption range of 200–900 nm in 10 mm × 10 mm quartz cuvettes. Fluorescence spectra were measured using a fluorescence spectrophotometer (Fluorolog3 with TCSPC; Horiba Ltd., Kyoto, Japan). The emission range of the sample was recorded from 200–900 nm with a fixed slit width (5 mm) in a 10 mm × 10 mm transparent cuvette. The zeta potential of the sample was recorded on a Zetasizer Nano ZS90 (Malvern Panalytical Ltd., Malvern, UK) with folded capillary zeta cell (DTS1070; Malvern Panalytical Ltd., Malvern, United Kingdom). Transmission electron microscopy (TEM) images were obtained using a transmission electron microscope (FEI Tecnai^TM^ G^2^ F20; FEI Company, Hillsboro, OR, USA). The samples were deposited on a carbon-coated Cu grid and observed using a transmission electron microscope operated at 300 kV. The Fourier transform infrared (FT-IR) spectra of the carbon dots were measured using an Alpha-P instrument (Bruker Corp.; Billerica, MA, USA).

### 2.3. Synthesis of Naphthalimide Probe 1

#### 2.3.1. Compound (**a**)

Compound (**a**) was synthesized using a condensation reaction between 4-aminonaphthalic anhydride (198 mg, 1 mmol) and 3-(dimethylamino)-1-propylamine (102 mg, 1 mmol) in 10 mL ethanol and refluxing for 6 h. Subsequently, the reaction mixture was cooled to 24 °C and a yellowish orange precipitate was separated. The crude product was washed with ethanol and then air-dried. Yield: 80%. ^1^H NMR in DMSO-d_6_ (δ = ppm): 8.67 (d, 1H), 8.50 (d, 1H), 8.2 (d, 1H), 7.8 (t, 1H), 7.5 (s, 2H), 6.89 (d, 1H), 4.02 (t, 2H), 2.3 (t, 2H), 2.02 (s, 6H), and 1.7 (t, 2H). ^13^C NMR in DMSO-d_6_ (δ = ppm): 164.2, 163.3, 134.3, 131.4, 130.1, 129.7, 124.4, 119.8, 108.6, 108.0, 57.3, 45.5, 26.3, and 19.0.

#### 2.3.2. Probe 1

The naphthalimide probe was synthesized by the nucleophilic substitution reaction of compound (**a**) (297 mg, 1 mmol) with methyliodide (216 mg, 1.5 mmol) in methanol (5 mL), and it required stirring for 7 h. After 7 h, the yellow-colored water-soluble precipitate was separated, washed with methanol, and dried in air. Yield: 78%, ^1^H NMR in DMSO-d_6_ (δ = ppm): 8.63 (d, 1H), 8.44 (d, 1H), 8.20 (d, 1H), 7.67 (t, 1H), 7.49 (s, 2H), 6.85 (d, 1H), 4.09 (t, 2H), 3.32 (t, 2H), 3.02 (s, 9H), and 2.09 (t, 2H), ^13^C NMR in DMSO-d_6_ (δ = ppm): 164.5, 163.5, 153.3, 134.5, 131.5, 130.3, 129.9, 124.4, 122.2, 119.8, 108.6, 107.9, 63.8, 52.6, 36.8, and 22.3.

### 2.4. Synthesis of Cationic Carbon Dots (CQDs)

Carbon dots were synthesized using a previously reported method [39]. Briefly, 100 mg of spermidine trihydrochloride was kept at 240 °C for 3 h. A dark black solid was dissolved in deionized (DI) water. The carbon dot solution was centrifuged at a relative centrifugal force of 20,000× *g* for 1 h. The resulting solid pellet was removed, and the supernatant was dialyzed against DI water through a dialysis membrane (molecular weight cut-off = 1.0 kDa) and vacuum-dried at 60 °C. The purified solution was stored at 4 °C.

### 2.5. Detection of HAase

In the control experiment, 50 μL (50 μg/mL) of the carbon dots, 50 μL (20 μg/mL) of HA, 5 μM of naphthalimide, and 20 U/mL of HAase were used in a PBS buffer at pH 7.4 for HAase detection. The resulting solution was incubated at 37 °C for a specific period of time (0–60 min), and fluorescence spectra were recorded for the quantitative analysis of HAase.

## 3. Result and Discussions

### 3.1. Synthesis and Characterization of Carbon Dots

**Figure 1:**
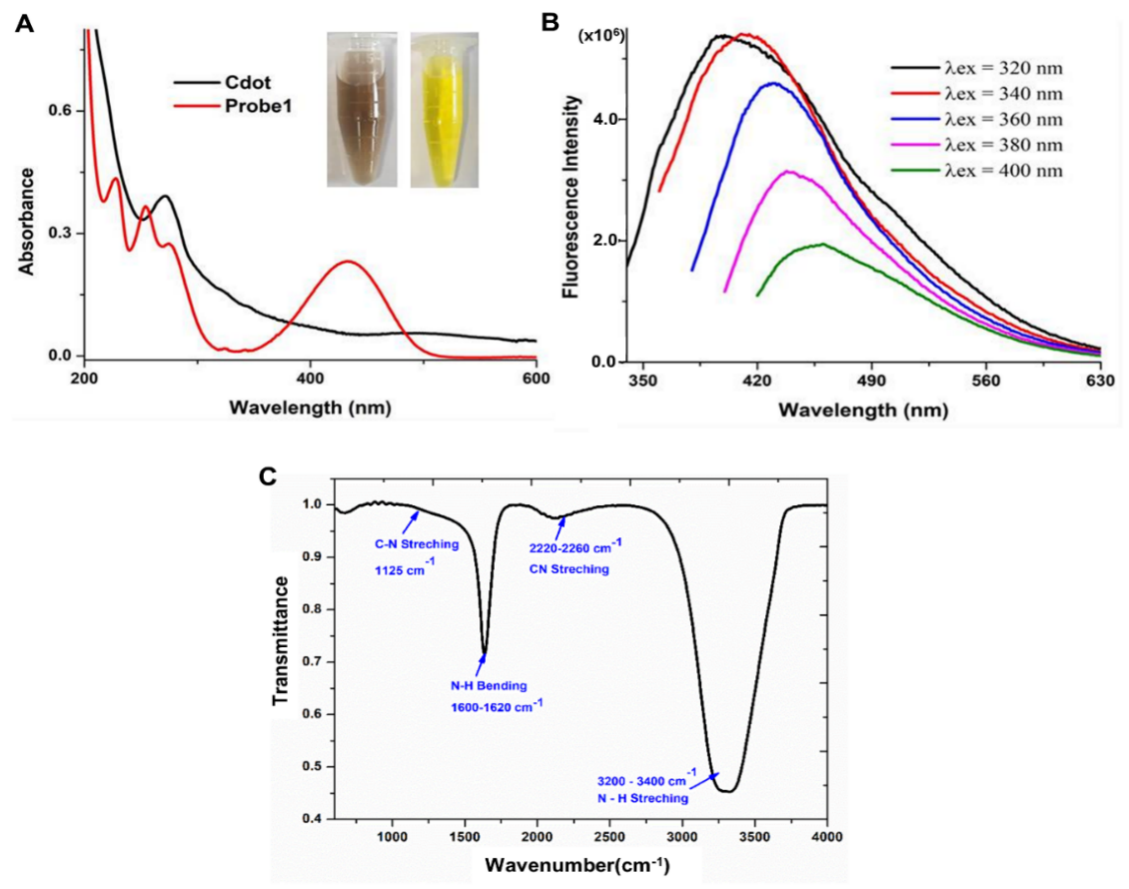
(**A**): UV-visible absorption spectra of carbon dots and probe 1 (10 μM) in an aqueous medium. (**B**): Excitation-dependent emission spectra of carbon dots in aqueous medium. (**C**): FTIR spectra of carbon dots

Cationic carbon dots were synthesized by the hydrothermal reaction of spermidine trihydrochloride and characterized by UV-visible absorption, emission, and TEM analysis. The UV-visible absorption spectra of the carbon dots show absorption bands (λ_abs_) at 270 nm, corresponding to the π–π* transition of the C=C bond, while a shoulder band at 300–450 nm corresponds to the n-π* transition of the C=O and C=N bonds, as shown in Figure 1A [40]. The excitation-dependent emission confirms the formation of carbon dots and is attributed to polycyclic clusters of different sizes, surface defects, and particles of different sizes, as shown in Figure 1B [41]. The TEM image studies show that the particles are spherical in shape and have sizes of 6–8 nm (Figure S1). The FT-IR spectra of the carbon exhibited vibration bands at 3200–3400, 2220–2260, and 1125 cm^−1^, corresponding to the N–H, C≡N, and C–N stretching vibrations, respectively. N–H bending was observed at 1600–1620 cm^−1^, as shown in Figure 1C. Thus, the FT-IR results clearly demonstrate that the carbon dots have a polyamine-like structure. It is well documented in the previous literature that carbon dots derived from quaternized ammonium surface precursors are positively charged or cationic in nature [42,43]. Hence, this assumption for positively charged carbon dots was confirmed by measuring the zeta potential of the synthesized carbon dots. The zeta potential (ζ) value of +19.5 mV confirmed the formation of stable cationic carbon dots (Figure S2).

### 3.2. Synthesis of Naphthalimide Probe 1

**Scheme 1:**
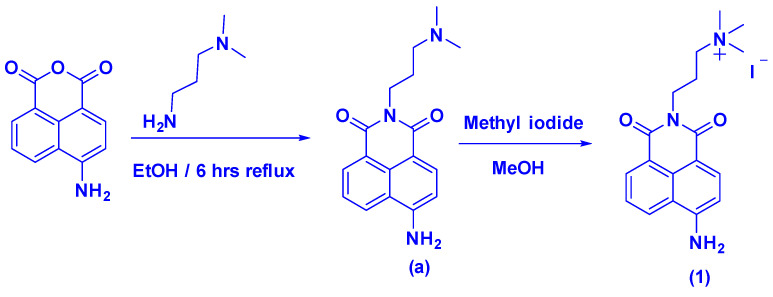
Synthesis of naphthalimide-based fluorescence probe 1.

Naphthalimide probe 1 was synthesized by a two-step reaction, that is, condensation followed by a nucleophilic substitution reaction (Scheme 1). The final product was characterized using NMR spectroscopy (Figures S3–S6). The NMR signal at 3.03 ppm, attributed to the 9H proton of the trimethyl group, confirms the formation of probe 1. The UV-visible absorption spectra of probe 1 (10 μM) in an aqueous medium exhibited prominent absorption peaks (λ_abs_) at 430 and 280 nm, which are assigned to the π–π* and n–π* transitions of the ligand, respectively, as shown in Figure 1A [44]. Probe 1 was excited (λ_ex_) at 430 nm and emitted (λ_em_) at 542 nm, as shown in Figure S7.

### 3.3. Sensing Mechanism

**Figure 2:**
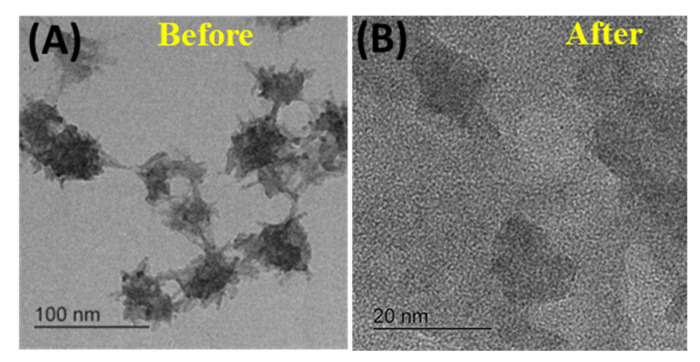
TEM images of sensor system, (**A**): before HAase addition, (**B**): after HAase addition (20 U/mL).

**Figure 3:**
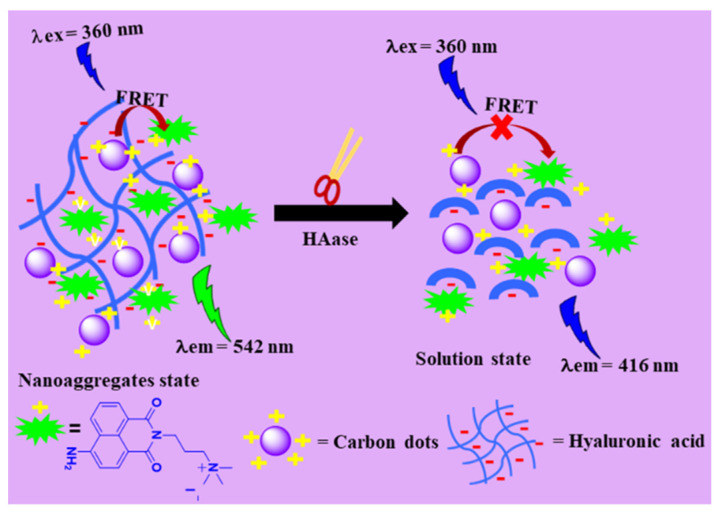
Graphical representation of the FRET mechanism for the sensing of hyaluronidase.

Two emission wavelength-based fluorescence sensors were designed to obtain a FRET mechanisam because the emission spectra of the carbon dots overlapped considerably with the absorption spectra of naphthalimide probe 1, as shown in Figure S8. The cationic carbon dot and naphthalimide probe were adsorbed on negatively charged HA through electrostatic interactions, and they formed a supramolecular nanoassembly. The supramolecular assemblies were characterized by TEM image analysis (Figure 2). The TEM image results show that the supramolecular nanoassembly changed from an aggregate state to a dispersion solution after treatment using HAase. The HAase enzyme selectively hydrolyzes the HA into small fragments and breaks down the supramolecular assemblies, leading to a disruption of the FRET mechanism, as shown in Figure 3.

### 3.4. Fluorescence Sensing

#### 3.4.1. Optimization of Experimental Conditions

**Figure 4:**
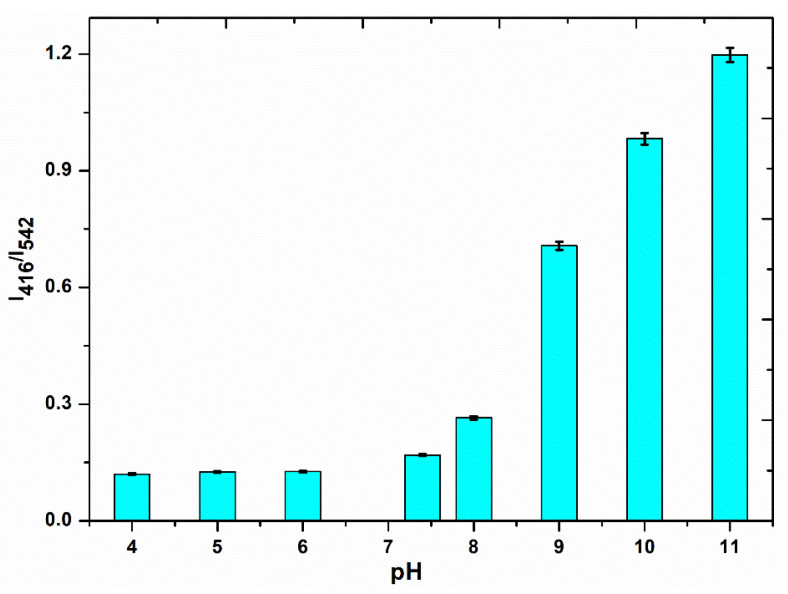
Effect of pH on the fluorescence intensity of the sensing system.

The optimization conditions of the sensor system were significantly affected by changes in pH and concentration of the fluorophore. Initially, a series of experiments were conducted to fix the concentration of the two fluorophores. Finally, 50 μL of carbon dots and 5 μM of naphthalimide probe 1 exhibited equal fluorescence intensities at 416 nm and 542 nm, respectively, indicating a good scope of ratiometric change. Accordingly, this concentration for the sensor was chosen for further studies. The effect of pH on the sensing probe was measured using fluorescence spectroscopy by changing pH from 4 to 11. The fluorescence intensity of the sensor remained unaltered at an acidic pH. Conversely, under basic conditions, the fluorescence intensity changed significantly, as shown in Figure 4. The fluorescence intensity of the carbon dots remained stable in both acidic and basic media, whereas naphthalimide probe 1 exhibited a decrease in fluorescence intensity with an increase in pH. Therefore, to confirm the stability of the sensor, we implemented all recognition studies in the PBS buffer (10 mM, pH = 7.4).

#### 3.4.2. Performance of Sensor System

**Figure 5:**
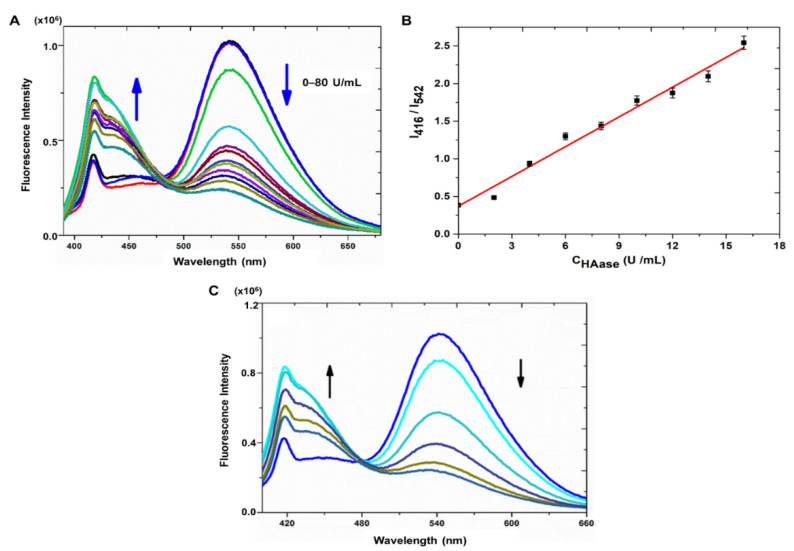
(**A**): Fluorescence titration experiment for sensor probe with HAase (0–80 U/mL) in phosphate buffered saline (PBS) buffer (10 mM, pH = 7.4). (**B**): Calibration plot between fluorescence intensity at I**_416_**/I**_542_** versus HAase concentration (0.1–16 U/mL). (**C**): Time-dependent fluorescence spectra of the sensor system in the presence of 20 U/mL of HAase.

The sensing ability of the sensor probe (50 μL of carbon dots, 50 μL of HA, and 5 μM of naphthalimide probe in PBS buffer at pH 7.4) for HAase detection was measured using fluorescence spectroscopy. The fluorescence spectra of the sensor system were excited at λ_ex_ = 360 nm and exhibited two emission wavelengths (λ_em_) at 416 nm, which was assigned to the carbon dots emission, and 542 nm, which was assigned to naphthalimide emission. The two emissions in the sensor system occurred owing to the adsorption of cationic carbon dots and the positively charged naphthalimide probe on the negatively charged HA through electrostatic interactions and the development of a FRET mechanism from the carbon dots to naphthalimide probe 1. Upon the addition of HAase to the sensing probe, the fluorescence intensity at 542 nm started to decrease, and the fluorescence intensity at 416 nm increased. This was owing to the hydrolysis of HA by the enzyme HAase, and because the FRET mechanism developed from carbon dots to naphthalimide was inhibited. Furthermore, an incremental quantity of HAase (0–80 U/mL) was added to the sensor system, and the fluorescence intensity was recorded. The fluorescence titration experiment showed that the sensor system exhibited a ratiometric response to the addition of HAase (Figure 5A). The calibration plot exhibited a linear relationship (R^2^ = 0.993) between the fluorescence intensity and the concentration of HAase (Figure 5B). The kinetics of the enzymatic reaction was investigated by recording the time-dependent fluorescence spectra of the sensor system in the presence of HAase (20 U/mL). The resulting solution was incubated at 37 °C for a specific period (0–60 min) and fluorescence spectra were then recorded to perform a quantitative analysis of HAase. The fluorescence spectra of the sensor system exhibited an increase in fluorescence intensity at 416 nm and a decrease at 542 nm with an increase in time, subsequently reaching a maximum at 60 min (Figure 5C). Further, to confirm complete enzymatic activity, the enzyme was incubated for 80 min for the fluorescence experiment. The detection limit was determined by the 3 sigma method. We obtained a value of 0.09 U/mL, which is less than the HAase level (0.26 ± 0.20 U/mL) found in normal human serum [45]. To make the proposed fluorescence assay useful in practice, further improvements are still needed; these will require further testing in complex fluid matrixes, such as serum and urine samples. The results should also be compared with thoes of traditional methods. Nevertheless, the above results suggest that the current approach is simple and highly sensitive for the detection of HAase compared with previous approaches reported in the literature (Table S1).

### 3.5. Selectivity Studies

**Figure 6:**
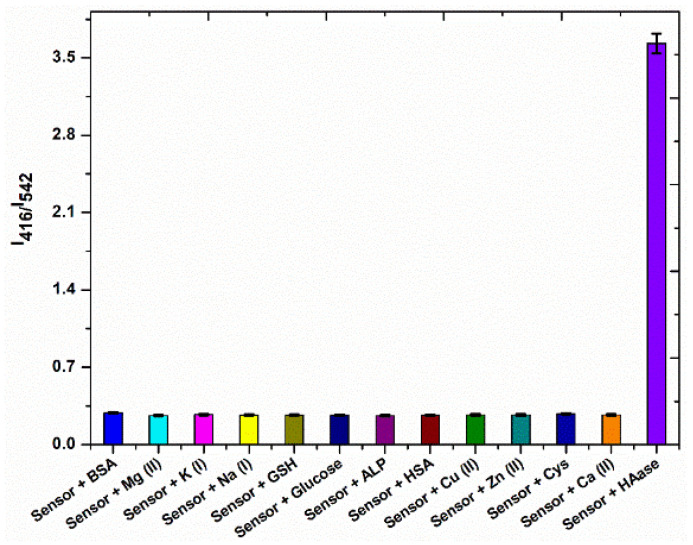
Fluorescence spectra of the sensor system in the presence of different competitive analytes (50 μM) in PBS buffer (10 mM, pH = 7.4).

The selectivity of the sensing probe for HAase detection was determined using potential interfering analytes, such as Mg (II), Ca (II), Zn (II), Cu (II), Na (I), K (I), BSA, HSA, glucose, Cys, and ALP (1 U/mL), under identical experimental conditions. Figure 6 shows that none of these competitive analytes exhibited changes in the fluorescence intensity of the sensing probe. HAase caused a considerable change in the fluorescence intensity (I_416_/I_542_) of the sensing probe, confirming the high selectivity of the sensor towards HAase detection.

## 4. Conclusions

We developed a simple, rapid, and sensitive fluorescence assay for the ratiometric detection of HAase in an aqueous medium. The FRET mechanism was successfully used to detect HAase. None of the competitive analytes interfered with the detection of HAase. A low detection limit of 0.09 U/mL was achieved, which is less than the HAase concentration in normal human serum. Moreover, this bioassay may find potential applications in the clinical diagnosis of HAase-related diseases.

## Data Availability

Data available on request to corresponding author.

## Supplementary Materials

The following are available online at https://www.mdpi.com/1996-1944/14/5/1313/s1, Figure S1: TEM image of carbon dot. (A) Low resolution TEM image; (B) high resolution TEM image. Figure S2: Zeta potential of carbon dots in 10 mM of PBS at pH 7.4. Figure S3: ^1^H NMR spectrum of compound (**a**) in DMSO-d_6_. Figure S4: ^13^C NMR spectrum of compound (**a**) in DMSO-d_6_. Figure S5: ^1^H NMR spectrum of probe 1 in DMSO-d_6_ Figure S6: ^13^C NMR spectrum of probe 1 in DMSO-d_6_. Figure S7: Emission spectra of naphthalimide probe 1 (10 μM) in water. Figure S8: Spectral overlap of carbon dot and probe 1. Table S1: Comparison between previous reported studies and the presented sensor
