# Supplementary material for: Carbon Dot/Naphthalimide Based Ratiometric Fluorescence Biosensor for Hyaluronidase Detection
Source: Materials (Basel). 2021 Mar 9;14(5):1313. doi: 10.3390/ma14051313 (PMC7967242; doi:10.3390/ma14051313)
Supplement: Supplementary file 1 [file materials-14-01313-s001.pdf]

Supplementary Materials for

Carbon Dot/Naphthalimide Based Ratiometric Fluorescence Biosensor for Hyaluronidase Detection

Pushap Raj [1], Seon-yeong Lee [2] and Tae Yoon Lee [1,2,*]

1 Department of Convergence System Engineering and Department of Biomedical Engineering, Chungnam National University, 99 Daehak-ro, Yuseong-gu, Daejeon 34134, Korea; pushap143@cnu.ac.kr
2 Department of Technology Education, Chungnam National University, 99 Daehak-ro, Yuseong-gu, Daejeon 34134, Korea; t.sylee29@gmail.com
* Correspondence: taeyoon.lee@cnu.ac.kr

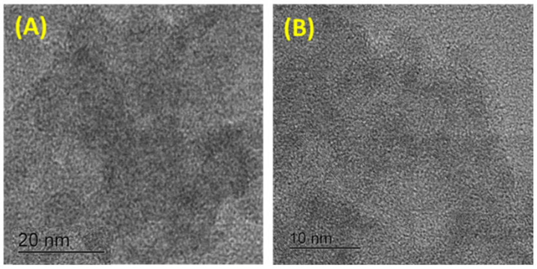

Figure 1. TEM image of carbon dot. (A) Low resolution TEM image; (B) high resolution TEM image.

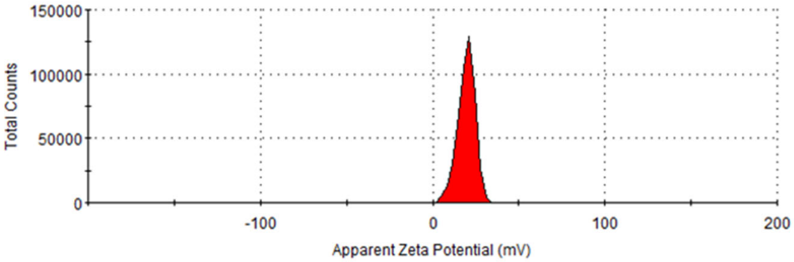

Figure S2: Zeta potential of carbon dots in 10 mM of PBS at pH 7.4.

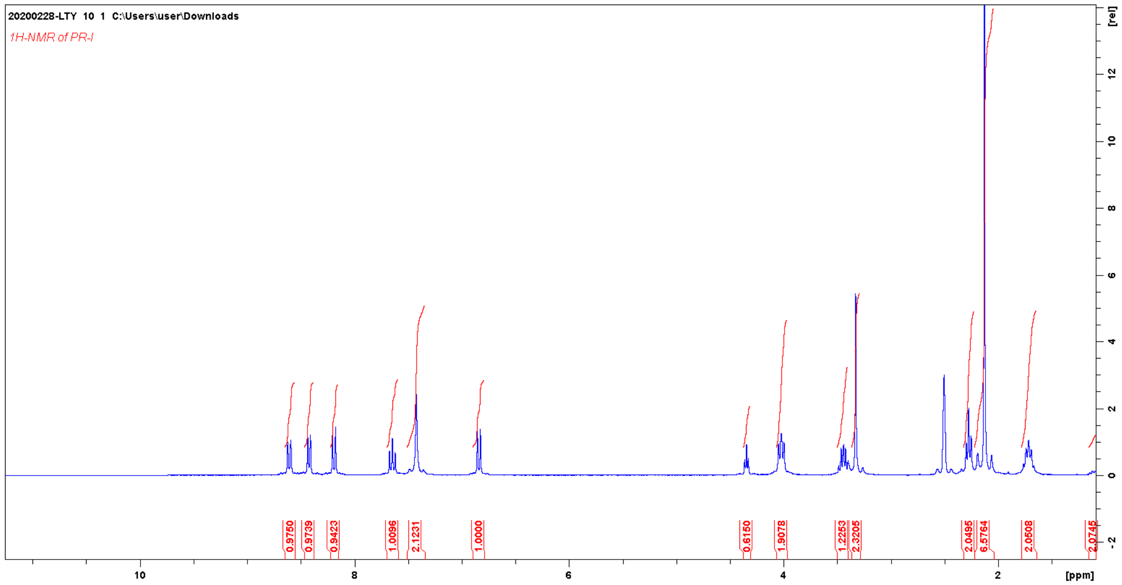

Figure S3: ^{1}H NMR spectrum of compound (a) in DMSO-d$_6$

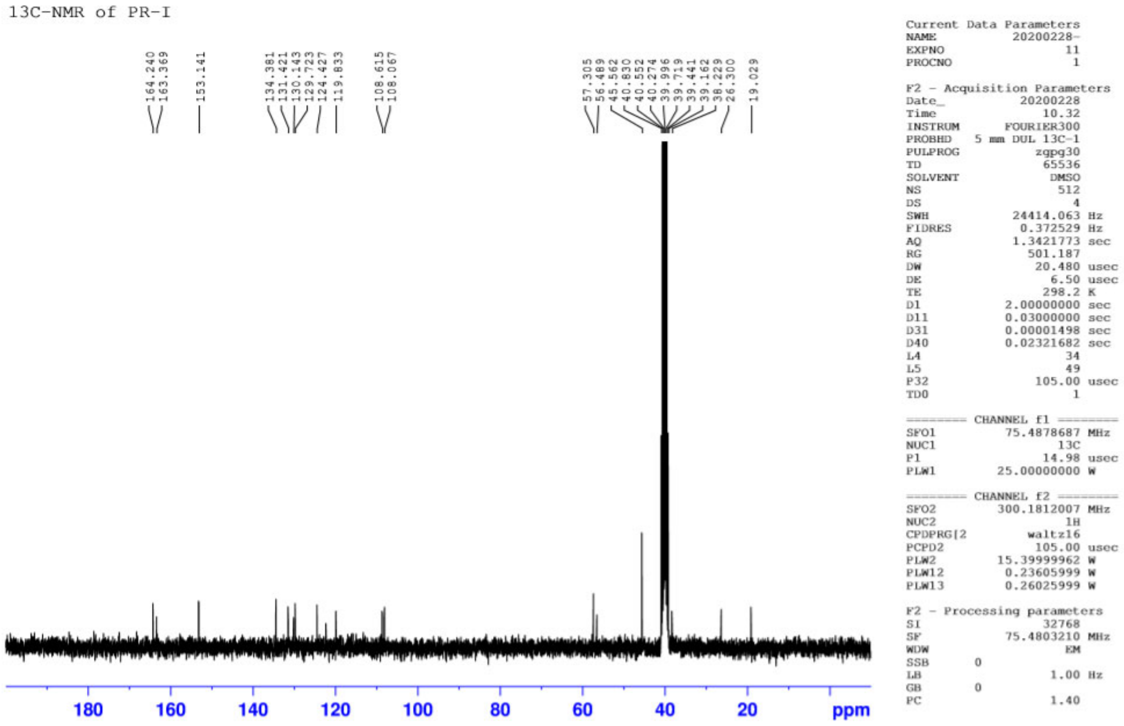

Figure S4: ^{13}C NMR spectrum of compound (a) in DMSO-d$_6$

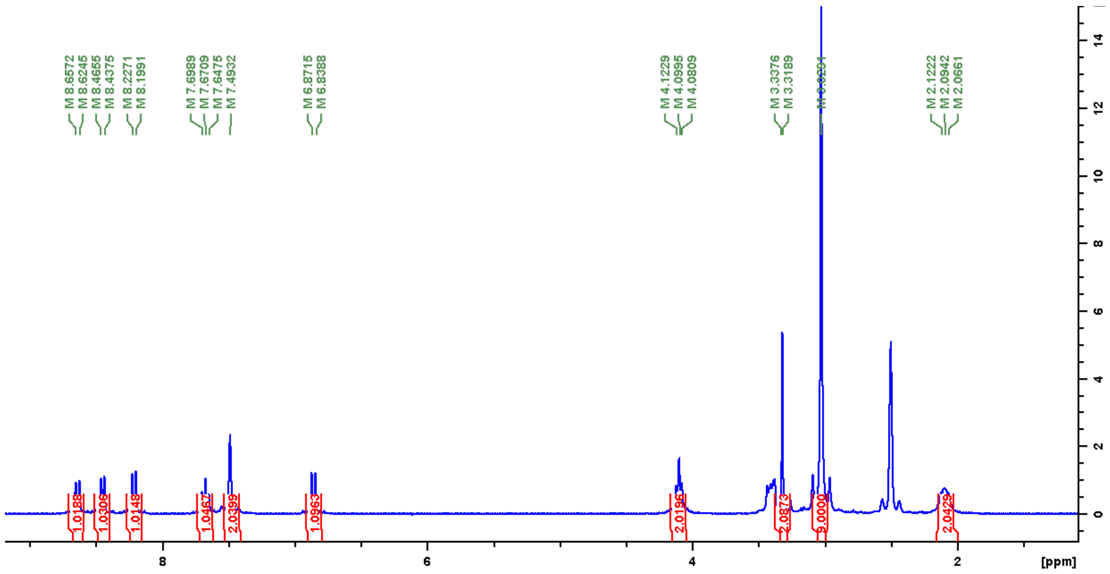

Figure S5: ^{1}H NMR spectrum of probe **1** in DMSO-d₆

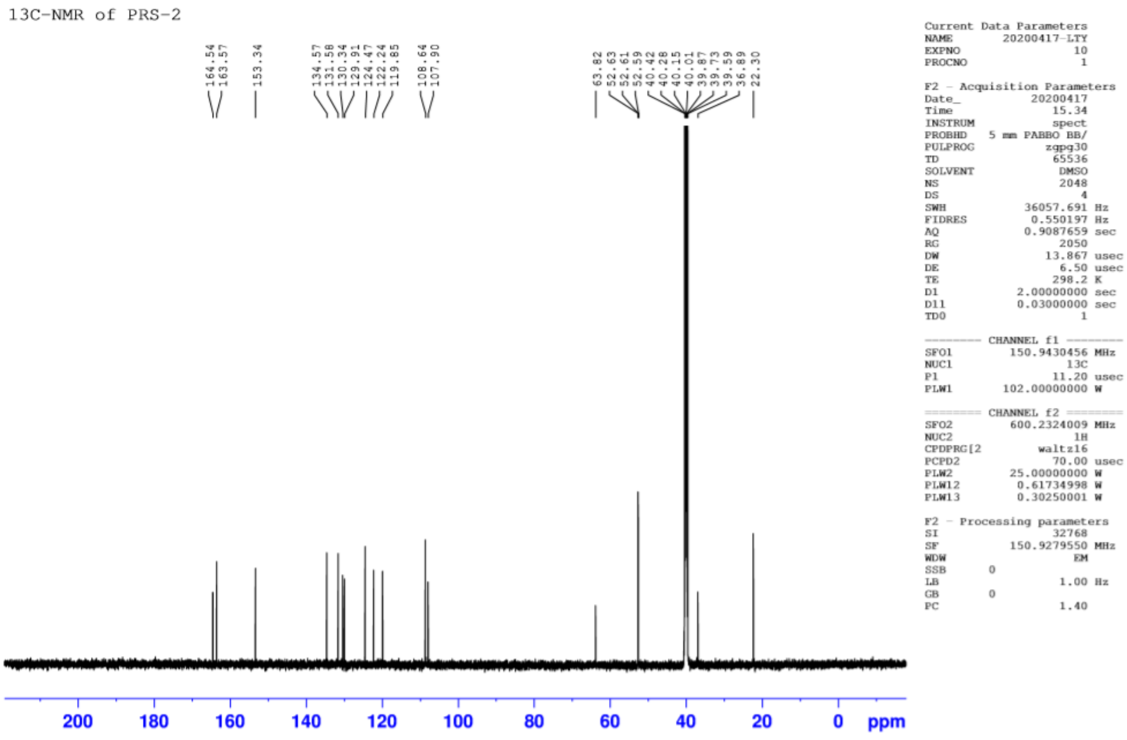

Figure S6: ^{13}C NMR spectrum of probe **1** in DMSO-d₆

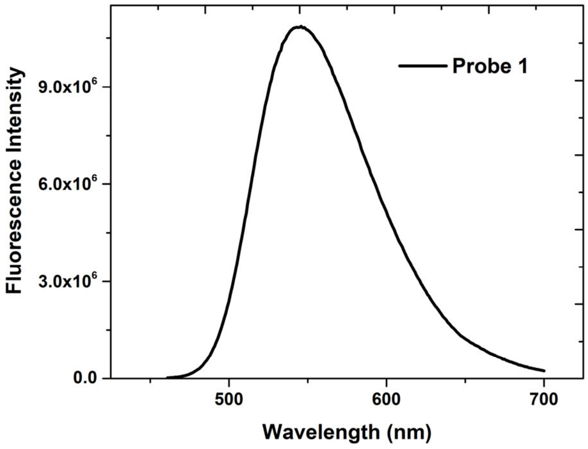

Figure S7: Emission spectra of naphthalimide probe **1** (10 μM) in water.

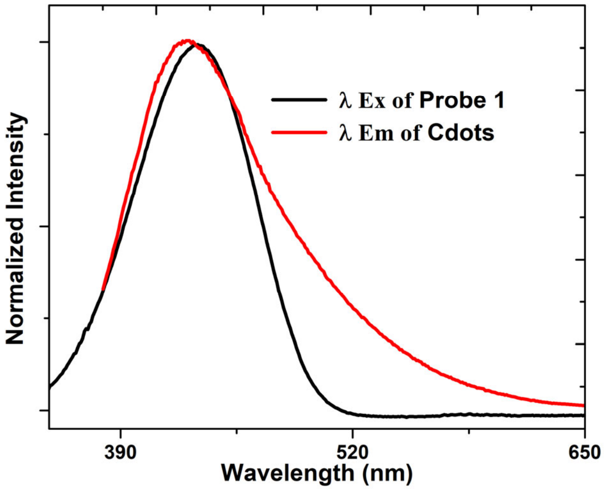

Figure S8: Spectral overlap of carbon dot and probe **1**

Table 1. Comparison between previous reported studies and the presented sensor.

Sensing system	Method	Fluorescence Intensity	Detection limit (U/mL)	Linear range (U/mL)	Reference
	Zymography		0.625	0.625–5	*Toxicon*, **2008**, 51, 1060-1067
Carbon dot	Fluorescence	Single wavelength	0.03 ng/mL	0–4	*Sensors and Actuators B: Chemical*, **2017**, 251, 503-508
Organic fluorophore	Fluorescence	Single wavelength	0.02	0.02–5	*Sensors and Actuators B: Chemical*, **2018**, 276, 95-100
Au/Ag nanocluster	Fluorescence	Single wavelength	0.3	0.5–37.5	*Sensors and Actuators B: Chemical*, **2019**, 282, 45-51
Organic fluorophore	Fluorescence	Ratiometric	0.007	N/A	*Biosensors and Bioelectronics*, **2016**, 79, 776–783

Si QDs/HA-δ-FeOOH nan assembly	fluorescence	Single wavelength	0.0127	0–12 ng/mL	*Biosensors and Bioelectronics,* **2020**,150, 1119-28
Up conversion Luminescence Nanoprobe	fluorescence	Ratiometric	0.6 ng/mL	0.9–150 ng/mL	*Anal. Chem.* **2015**, 87, 5816−5823
Carbon-dot/NR assembly	fluorescence	Ratiometric	0.05	0.1–8	*Anal. Chem.* **2017**, 89, 8384−8390
Core−Shell HA-AuNPs @SiNPs Nanoprobe	Fluorescence	Single wavelength	0.03	0.01–10	*ACS Sustainable Chem. Eng.* **2018**, 6, 16555−16562
Perylene self-assembly	Fluorescence	Single wavelength	0.03	0.1–10	*New J. Chem.,* **2019**,43, 3383-3389
Carbon dot/ chitosan@AuNPs	Fluorescence and Colorimetric	Single wavelength	0.27	2–70	*Luminescence.,* **2020**, 3, 43–51.
MoS_2 QDs	Fluorescence	Single wavelength	0.7	1–50	*ACS Appl. Mater. Interfaces,* **2016**, 8, 11272
Carbon dot/ naphthalimide nanoassembly	Fluorescence	Ratiometric	0.09	0.1–80	*Present work*
